# Evolutionary and co-evolutionary phage training approaches enhance bacterial suppression and delay the emergence of phage resistance

**DOI:** 10.1093/ismeco/ycae082

**Published:** 2024-06-12

**Authors:** Lyman Ngiam, Karen Weynberg, Jianhua Guo

**Affiliations:** Australian Centre for Water and Environmental Biotechnology, The University of Queensland, 4 Gehrmann Laboratories Building, Research Road, St Lucia, Queensland 4072, Australia; Australian Centre for Ecogenomics, The University of Queensland, Chemistry Building 68, Cooper Road, St Lucia, Queensland 4072, Australia; Australian Centre for Water and Environmental Biotechnology, The University of Queensland, 4 Gehrmann Laboratories Building, Research Road, St Lucia, Queensland 4072, Australia

**Keywords:** bacteriophage, evolution, evolutionary, co-evolutionary, phage training, virus host interaction, bacteriophage-resistant bacteria mutant

## Abstract

The development of phage resistance by bacteria is a major barrier that impedes the therapeutic use of phages. Phage training has been proposed as a novel tool that harnesses the evolutionary potential of phages to improve phage infectivity. Both evolutionary and co-evolutionary phage training models have been previously reported to train phages. However, both of these phage training models have been reported able to effectively suppress the emergence of phage-resistant bacteria mutants, thus presenting a contradictory phenomenon. Therefore, in this study, we set out to systematically compare the effectiveness of both evolutionary and co-evolutionary phage training models with regard to phage physiology, infectivity, and genotype. To this end, a natural lytic phage capable of infecting a *Klebsiella pneumonia* strain was isolated from wastewater and subjected to evolutionary and co-evolutionary phage training for 30 days. After the phage training, the physiology and genomic characteristics of evolved and co-evolved phages were assessed. Our results demonstrated that both evolved and co-evolved phages exhibit improved bacterial suppression activity and are able to delay the emergence of phage resistance. Furthermore, both phages harbored unique genome mutational changes in different functionally associated phage proteins. Similarly, evolved and co-evolved phage-resistant bacteria mutants that arose post phage infection displayed varying phage resistance sensitivities, which may be correlated to the unique genome mutational change identified in cell membrane structure. In particular, co-evolved phage-resistant bacteria mutants exhibited less phage resistance compared to evolved phage-resistant bacteria mutants. These results highlighted the finding that the co-evolutionary phage training model serves as a better phage training model as it endows phage with improved infectivity, but also selects for phage-resistant bacteria with a lower phage resistance when compared to evolutionary phage training.

## Introduction

The development of phage resistance is a commonly observed phenomenon found in bacteria after phage infection [1]. Phage resistance is also one of the major limitations associated with the therapeutic use of phages against antibiotic-resistant bacteria. However, the development of phage resistance by bacteria could be overcome by phage training. Specifically, as a virus specific to bacteria, a phage is able to evolve itself to adapt and re-infect phage-resistant bacteria. The reciprocal adaptation and counter-adaptation interactions between phages and bacteria are among the key factors identified that drive the evolution of diverse microbial communities [2, 3].

Phage training is an alternative tool to enhance the infectivity or extend the host range of desired phage candidates. In this scenario, the selected phage candidate is “pre-adapted” to effectively infect the target bacteria host whilst preventing or delaying the emergence of phage-resistant bacteria mutants [4–6]. To date, two phage training models have been reported in the literature, namely evolutionary and co-evolutionary phage training. The main differences between these two models are that the target bacterium in the evolutionary phage training model remains the same, whereas the target bacterium in the co-evolutionary phage training model is constantly co-evolving with its phage counterpart to escape phage predation. Although both phage training models have been previously described [4, 5, 7], there is a lack of systematic comparisons of effectiveness of different phage training models for preventing or delaying the emergence of phage-resistant bacteria.

In this study we aimed to compare and assess how different phage training models affect phage physiology, genotype, and effectiveness in preventing and/or delaying the emergence of phage-resistant bacteria. To this end, a natural lytic phage infecting *Klebsiella pneumoniae* strain, a model multidrug resistant bacterium, was isolated, plaque purified, and subjected to two different phage training models for a total of 30 days. After phage training, the infectivity, phenotypic, and genomic features of evolved and co-evolved phages were assessed in comparison to the ancestral phage. Our study highlights the findings that both evolutionary and co-evolutionary phage training models varyingly improve phage physiology and infectivity. Further time-shift assays using phage-resistant bacteria mutants generated using evolved and co-evolved phage populations revealed that co-evolved phages can better delay the emergence of phage-resistant bacteria.

## Materials and methods

### Bacterial strains and culture conditions

In this study, *K. pneumoniae* 52 145 belonging to serotype O1:K2, sequence type 66, was selected as the bacterial host for phage isolation. This bacteria strain was selected because it presents as a model multidrug-resistant bacterium. The bacterial strain was cultivated in Luria-Bertani liquid broth medium (LB) or LB with 1% agar solid medium and incubated at 37°C.

### Bacteriophage isolation, purification, and propagation

Lytic bacteriophages were isolated using domestic wastewater collected from a full-scale wastewater treatment plant in Brisbane, Australia. The procedures for phage isolation, purification, and propagation were performed as previously described [8, 9].

### Transmission electron microscopy

Transmission electron microscopy was performed to visualize the morphology of the isolated phages according to the protocol described previously [8, 9]. Briefly, an aliquot of purified phage lysate (2 μl) was dropped onto a 200-mesh carbon-coated copper grid, negatively stained with 1% (wt/vol) uranyl acetate, and air dried. Next, the grid was examined using a transmission electron microscope (JEM-1011, JEOL Ltd. Tokyo, Japan) with accelerating voltage of 80 kV.

### Evolutionary and co-evolutionary phage training approaches

**Figure 1:**
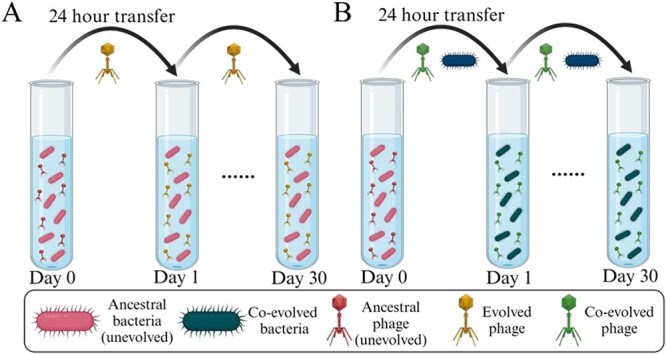
Phage experimental evolution design and proposed bacterial response in this study. (A) Evolutionary phage training model where phage is subjected to evolve continuously on ancestral bacteria host. Prior to each transfer, the crude lysates are filtered with 0.22-μm polyethersulfone membrane filter to remove potential bacteria contamination, keeping only the phage filtrate. (B) Co-evolutionary phage training model where phage and bacteria co-evolve together. In each transfer, the microcosm community containing both phage and bacteria are directly transferred into new LB medium. Created with BioRender.com.

A naturally occurring lytic phage infecting *K. pneumoniae* bacteria in this study was isolated and purified. The bacteria-killing assay profile of this phage demonstrated that it can suppress the growth of bacteria only for an average of 6.03 ± 0.03 hours. Thereafter, the emergence of phage-resistant bacteria as indicated by an increase in the optical density (OD) value was recorded. Therefore, the demonstrated temporal inhibition of bacterial growth by this phage makes it an ideal candidate to assess the effects of phage training to prevent or delay the emergence of phage-resistant bacteria mutants. Here, two types of phage training, namely evolutionary and co-evolutionary phage training models, were conducted for a total period of 30 days, as shown in Fig. 1. For the evolutionary phage training model (Fig. 1A). The phage was subjected to continuous evolution on an ancestral bacteria host, whereas for the co-evolutionary phage training model (Fig. 1B), both the phage and bacteria were continuously co-evolving. For both models phage training was conducted in a 96-well plate. At day 0 of the experiment setup, 100 μl of phage suspended in saline magnesium buffer solution (~10^9^ PFU/mL) and 100 μl of fresh bacteria inoculum diluted overnight in LB medium (OD_600_ = 0.3, ~10^8^ colony forming units [CFU]/ml) were initially loaded per well in the 96-well plate. The plate was then incubated at 37°C with shaking at 120 rpm overnight. For the evolutionary phage training model, after 24 hours of incubation phage lysates from the plate well were collected, centrifuged, and filtered using a 0.22-μm polyethersulfone filter membrane. Next, 100 μl of the phage filtrates were transferred into new wells containing 100 μl diluted fresh bacteria inoculum (OD_600_ = 0.3, ~10^8^ CFU/ml). Meanwhile, for the co-evolutionary phage training model, after 24 hours of incubation, 100 μl of the microcosm community (containing both phage and bacteria) were transferred into new wells containing 100 μl of fresh LB medium. Both evolutionary and co-evolutionary phage training models were performed in biological triplicate. Details of the biological triplicate design for both evolving and co-evolving phage training models in a 96-well plate are shown in Fig. S1.

### *In vitro* bacterial killing assay of evolved and co-evolved phage populations

To assess the infectivity of phages towards bacterial hosts, *in vitro* bacterial killing assays were performed by measuring the OD_600nm_ growth profile of bacteria after exposure to ancestral, evolved, and co-evolved phages. Specifically, evolved and co-evolved phage population lysates collected from days 0, 10, 20, and 30 were compared against the ancestral phage. Prior to the *in vitro* bacterial killing assays, titers of the collected evolved and co-evolved phage population lysates were assessed via plaque assays. During the *in vitro* bacterial killing assays performed in a 96-well plate, a phage-to-bacteria ratio of MOI (multiplicity of infection) 10 was introduced per well (100 μl phage solution, ~10^9^ PFU/ml) and 100 μl diluted overnight fresh bacteria inoculum in LB medium (OD_600_ = 0.3, ~10^8^ CFU/ml). Next, OD_600nm_ was monitored for 24 hours using a CLARIOstar® *Plus* multi-mode microplate reader (BMG Labtech, Offenburg, Germany).

### Isolation and purification of evolved and co-evolved phages from a single-phage population

Before phage training, single phage plaques from single day 30 evolved and co-evolved phage populations were isolated and purified for further phage phenotypic and genomic characterization as previously described [8] with some modifications. Briefly, prior to each round of plaque purification assay, the infectivity of the isolated phage was verified through a phage killing assay against the ancestral bacteria host to ensure a consistent infectivity pattern.

### Phage population dynamics of single plaque purified evolved and co-evolved phages

The determination of phage adsorption and one-step growth curve was carried out as previously described [8] with some modifications. Briefly, the phage adsorption and one-step growth curve assays were conducted using an MOI of 0.1. For phage adsorption, a phage–host suspension of 1 ml volume was sampled every 5 min, for a total period of 20 mins. For phages displaying prolonged adsorption, the total monitoring period was extended to 40 mins. For the one-step growth curve, the supernatants were discarded after centrifugation and the pellets were resuspended in 30 ml fresh LB medium and incubated with shaking at 37°C. The burst size of the phage was calculated by dividing the phage titres at the plateau phase by the initial number of infected bacterial cells as previously determined from phage adsorption assays.

### Resistance dynamics of phage-resistant mutants against evolved and co-evolved phage populations

Evolved and co-evolved phage-resistant bacteria mutants from different time points (day 0, 10, 20, and 30) were generated to assess their resistance dynamics against evolved and co-evolved phage populations. Ten phage-resistant bacteria mutant colonies grown in the presence of evolved or co-evolved phage populations from different time points were randomly picked and grown overnight in LB medium at 37°C. These overnight cultures were then centrifuged (8000 g for 10 min, 25°C), washed with PBS buffer twice and plated to obtain single colonies. This process was further repeated for an additional two times to ensure no phage contamination. In total, 80 phage-resistant bacteria mutant isolates were generated (40 phage-resistant bacteria mutant isolates generated using evolved phage populations, and 40 phage-resistant bacteria mutant isolates generated using co-evolved phage populations from each time point). Next, the resistance dynamics of the phage-resistant bacteria mutant isolates were tested against evolved and co-evolved phage populations using a phage spot assay.

### Phage DNA extraction and genome sequencing of phage

The extraction of phage DNA was carried out using a phage DNA isolation kit (Norgen Biotek Corp., Thorold, Canada) according to the manufacturer’s protocol. Both a Qubit fluorometer (Thermo Fisher Scientific) and Nanodrop (Nanodrop Technologies, USA) were used to assess the quantity and quality of phage DNA prior to sequencing. Next, the extracted phage DNAs were submitted to Novogene for whole genome sequencing using NextSeq (Illumina) genome sequencing (1 Gbp sequencing depth) as well as Nanopore sequencing (48-hour run). Sequence data returned in raw FASTQ format (Illumina) and FAST5 format (Nanopore) were quality checked, trimmed, and hybrid assembled using Unicycler v0.4.9. Full details for processing raw Illumina and Nanopore sequencing data prior to assembly are described in Supplementary Text S1.

### Bioinformatic analysis of phage genome sequence

The resulting hybrid assembled phage complete draft genomes from Unicycler were annotated as previously described [8] with slight modifications. Briefly, whole genome comparison of the isolated phages in this study and related phage genomes available at the NCBI database were visualized using the Basic Local Alignment Search Tool (BLAST) Ring Image Generator (BRIG) [10]. Mutational changes associated with single nucleotide polymorphisms (SNPs) and indel variants in evolved and co-evolved phages were performed using Geneious plugins FreeBayes tool [11].

### Whole genome sequencing of ancestral, evolved and co-evolved phage-resistant bacteria mutants

Phage-resistant bacterial mutants were generated, and genome analysed as previously described [8] with slight modifications. Briefly, raw reads generated from sequencing were paired end, quality checked, and trimmed using Geneious Prime 2023.2.1 (https://www.geneious.com). Next, the processed reads were assembled by mapping against closely related reference genomes available at the NCBI database (*K.pneumoniae* strain 52 145 FO834906) using the Geneious plugins BowTie2 tool [12]. SNPs and indel variants were performed using the Geneious plugins FreeBayes tool [11].

### Statistical analysis

All experiments were performed in triplicate when required, and the values were reported in the format of mean ± standard deviation. One-way analysis of variance using multiple Dunnett’s test was used for determination of statistical significance using Graph Pad Prism 6.05 software. A *P* value <.05 was considered as statistically significant.

### Data availability

The complete sequences of ancestral phage vB_KpS_KW1, evolved phage vB_KpS_KW1.1 and co-evolved phage vB_KpS_KW1.2 were individually submitted in the NCBI database under the accession numbers OP978314, OP978315, and OP978316, respectively. For phage-resistant bacteria mutants, the accession numbers can be found in Bioproject accession number PRJNA1086631.

## Results

### Phage morphology

In this study, a lytic phage infecting *K. pneumoniae* 52 145 strain was isolated and named as vB_KpS_KW1 (herein referred to as phage KW1). Next, phage KW1 was subjected to both evolutionary and co-evolutionary phage training for 30 days (Fig. 1A and B). After phage training, single day 30 evolved and co-evolved phages were isolated, plaque purified, and named vB_KpS_KW1.1 (herein referred to as phage KW1.1) and vB_KpS_KW1.2 (herein referred to as phage KW1.2), respectively. Visualisations obtained with transmission electron microscopy of phages KW1, KW1.1, and KW1.2 present *Siphovirus*-like morphology, with average size of 251.3 ± 1.5 nm (Supplementary Fig. S14).

### Phage population dynamics of ancestral, evolved, and co-evolved phages

Phage population dynamics in terms of adsorption and one-step growth curve were conducted to assess the population dynamics of the evolved phage KW1.1 and the co-evolved phage KW1.2 in comparison to ancestral phage KW1, and the results are shown in Table 1. For ancestral phage KW1, the average maximum phage adsorption of 52.1% ± 2.5% was achieved in 12 mins. For the evolved phage KW1.1, average maximum phage adsorption of 68.2% ± 3.1% was achieved in 30 minutes, whereas the co-evolved phage KW1.2 achieved average maximum phage adsorption of 55% ± 1.1% in 35 min.

**Table 1:** Population dynamics of original, evolve and co-evolve phage.

Phage	Phage adsorption assay		Phage one-step growth curve	
	Maximum phage adsorption period (mins)	Maximum phage adsorption (%)	Latent period (mins)	Burst size (phage per cell)
KW1 (unevolved)	12	52.1% ± 2.5%	20	100 ± 6
KW1.1 (evolved)	30	68.2% ± 3.1%	10	127 ± 5
KW1.2 (co-evolved)	35	55% ± 1.1%	10	74 ± 5

Each value is presented as mean values ± standard deviation from three independent experiments.

Meanwhile, the one-step growth curves of the phages were also different. The ancestral phage KW1 was found to have an average latent period of 20 min, followed by average burst size of 100 ± 6 phages per cell. The evolved phage KW1.1 and the co-evolved phage KW1.2 were found to have similar average latent period of 10 min but differ in terms of burst sizes (average burst sizes of 127 ± 5 and 74 ± 5 phage per cell, respectively).

### Evolved and co-evolved phage populations *in vitro* killing assay from different time points

**Figure 2:**
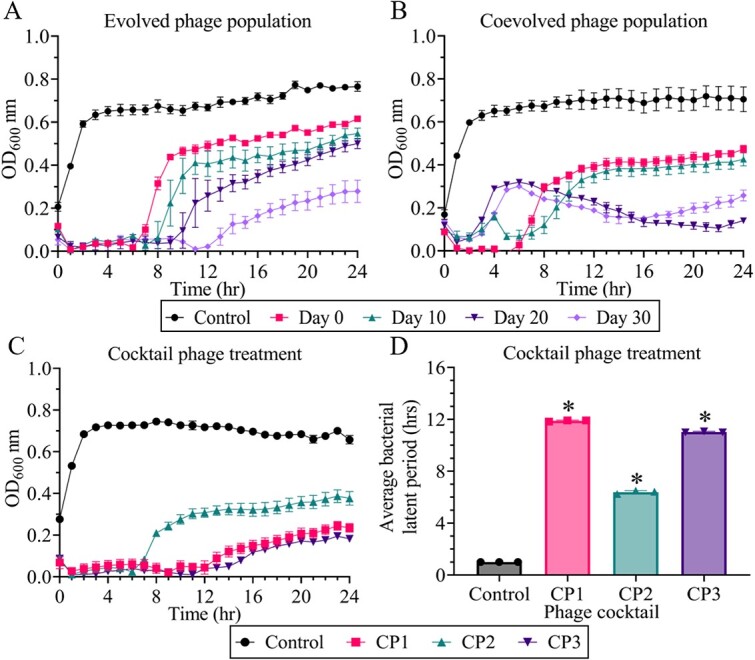
Phage killing assay against *K. Pneumoniae* 52 145 strain. (A) Phage killing assay of evolved phage population; (B) phage killing assay of co-evolved phage population; (C) phage cocktail-based killing assay infecting *K. Pneumoniae* 52 145 strain using CP1 (phage KW1 + phage KW1.1), CP2 (phage KW1 + phage KW1.2) and CP3 (phage KW1.1 + phage KW1.2). (D) Statistical analysis of bacterial suppression period exerted by different cocktail-phage treatments. Each value is presented as mean values ± standard deviation from three independent experiments. *P < .05; statistically significant suppression of bacteria growth compared to the control, which consists of only bacteria.

The *in vitro* killing assays of evolved and co-evolved phage populations from different time points were assessed and compared against the ancestral phage KW1. Specifically, the infectivities of evolved and co-evolved phage population lysates at day 0, day 10, day 20, and day 30 were assessed. As shown in Fig. 2A and B, the infectivity profiles of both evolved and co-evolved phage populations showed different infectivity patterns when compared to the ancestral phage.

The ancestral phage KW1 was able to suppress bacteria growth for ~6 hours. For the evolved phage populations, the infectivity was observed to improve in a time-dependent manner. In particular, the bacteria growth suppression period exerted by evolved phage populations extended from 6 to 12 hours after 30 days of phage training (Fig. 2A). Meanwhile, the co-evolved phage populations exhibited different infectivity profiles compared to the evolved phage populations. The co-evolved phage population from day 10 was found to have an extended bacteria suppression period of ~7 hours (Fig. 2B). Meanwhile, the co-evolved phage populations from day 20 and day 30 exhibited similar infectivity profiles during which partial lysis of the bacteria host was observed within the first 6 hours, followed by complete lysis of the bacteria host for the remaining 24 hours.

### *In vitro* killing assay of cocktail phage

Although extended bacterial suppressions were observed with the evolved and co-evolved phage populations, the presence of phage-resistant bacteria mutants could still be observed. As such, cocktail-based phage treatments were designed to compare the infectivity efficiency of cocktail-based and single phage-based phage treatments. Three different cocktail-based phage treatments consisting of the ancestral phage KW1, the evolved phage KW1.1, and the co-evolved phage KW1.2 were formulated and compared against the control group with no phage treatment. From the results (Fig. 2C and D), cocktail phage treatment 1 (CP1) consisting of the ancestral phage KW1 and the evolved phage KW1.1 displayed the longest bacteria growth suppression period (average 11.9 ± 0.05 hours, *P* < .05). Cocktail phage treatment 2 (CP2) consisting of the ancestral phage KW1 and the co-evolved phage KW1.2 was found to exert the lowest average bacterial suppression period (average 6.4 ± 0.13 hours, *P* < .05). Cocktail phage treatment 3 (CP3) consisting of the evolved phage KW1.1 and co-evolved phage KW1.2 was found to exert average bacteria growth suppression period of 11 ± 0.12 hours (*P* < .05).

### Phage-resistant bacteria mutant resistance dynamic against evolved and co-evolved phage populations

**Figure 3:**
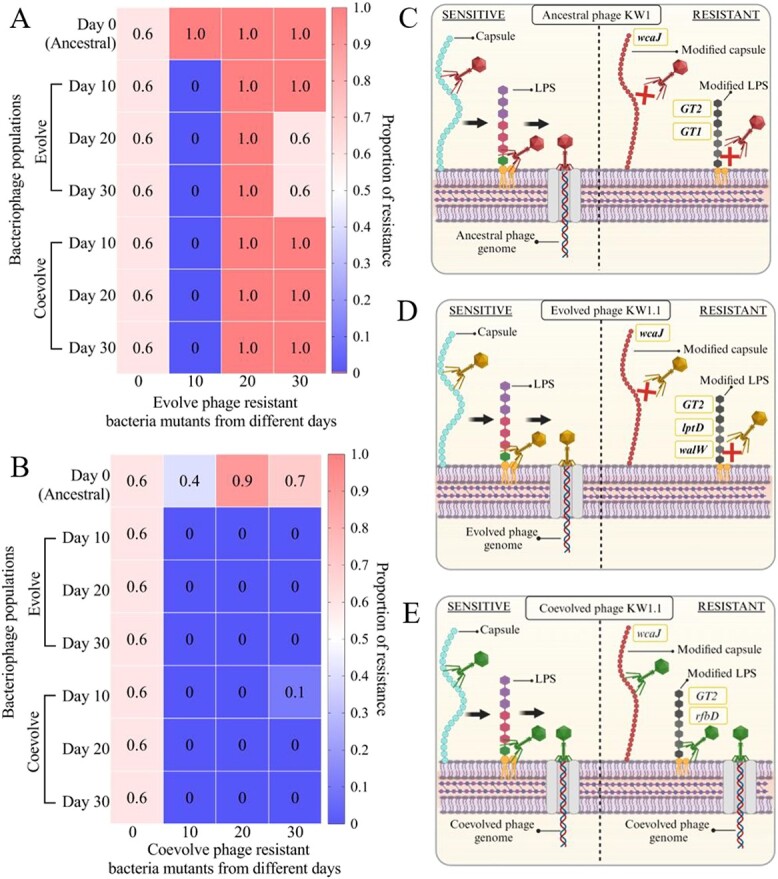
Sensitivity analysis of phage-resistant bacteria mutants and the identified genes responsible for conferring phage resistance. (A) Sensitivity analysis of evolved phage-resistant bacteria mutants generated using ancestral phage (day 0 mutants) and evolved phage populations from day 10, 20 and 30. (B) Sensitivity analysis of co-evolved phage-resistant bacteria mutants generated using ancestral phage (day 0) mutants and co-evolved phage populations from day 10, 20 and 30. (C) Mutations identified in phage ancestral phage KW1 resistant bacteria mutant conferring phage resistance. (D) Mutations identified in evolved phage KW1.1 phage-resistant bacteria mutant conferring phage resistance. (E) Mutations identified in co-evolved phage KW1.2 resistant bacteria mutant conferring partial resistance. Created with BioRender.com.

The infectivity dynamics of evolved and co-evolved phage populations against phage-resistant bacteria mutants were also assessed. Phage-resistant bacteria mutants generated using the ancestral phage KW1 (day 0 mutants) were found to exhibit the same proportion of phage resistance (6 out of 10 isolates) against day 10, 20, and 30 evolved and co-evolved phage populations (Fig. 3A and B).

For the evolved phage-resistant bacteria mutants (Fig. 3A), the day 10 evolved phage-resistant mutants exhibited complete phage resistance against ancestral phage KW1 but were completely sensitive to all other tested evolved and co-evolved phage populations. Conversely, day 20 evolved phage-resistant bacteria mutants were found to exhibit complete phage resistance against ancestral phage KW1 and all other evolved and co-evolved phage populations. Day 30 evolved phage-resistant bacteria mutants were also completely resistant to all other tested phages with partial phage resistance against day 20 and 30 evolved phage populations (6 out of 10 isolates).

For the co-evolved phage-resistant bacteria mutants (Fig. 3B), day 10 co-evolved phage-resistant bacteria mutants were shown to have partial phage resistance against ancestral phage KW1 (4 out of 10 isolates) but were completely sensitive to all other evolved and co-evolved phage populations. The day 20 co-evolved phage-resistant bacteria mutants exhibited complete resistance to ancestral phage KW1 (10 out of 10 isolates) and were completely sensitive to all other evolved and co-evolved phage populations. Similarly, day 30 co-evolved phage-resistant bacteria mutants were completely sensitive to all tested phages, except for ancestral phage KW1 (8 out 10 isolates) and day 10 co-evolved phage populations (1 out of 10 isolates).

### Genome characteristics of evolved and co-evolved phage in comparison to ancestral phage

After phage training, the genomes of the evolved phage KW1.1 and the co-evolved phage KW1.2 were extracted and analysed in comparison to the ancestral phage KW1. The genomes of the evolved phage KW1.1 and the co-evolved phage KW1.2 shared similar genome characteristics to the ancestral phage KW1 in terms of sequence length, guanine-cytosine content, and the number of open reading frames found.

Additionally, whole genomes of phages KW1, KW1.1, and KW1.2 were BLAST against available phage collection databases deposited at NCBI. The BLAST search results were visualized using BRIG (Supplemental Fig. S15). The results showed that phages KW1, KW1.1, and KW1.2 displayed the highest similarity (coverage 94%, percentage identity 94.96%–95.26%) to two other phages: *Klebsiella* phage mtp19 (OX335422.1) and *Klebsiella* phage NJS2 (NC_048043.1). In conjunction with whole genome comparison, a phylogenetic tree based on a phage terminase large subunit protein was also constructed to determine the taxonomy of phages in this study. From the result (Fig. S16), phages KW1, KW1.1 and KW1.2 were found clustered within the same phage genus, *Webervirus*, under the phage family *Drexlerviridae.*

**Figure 4:**
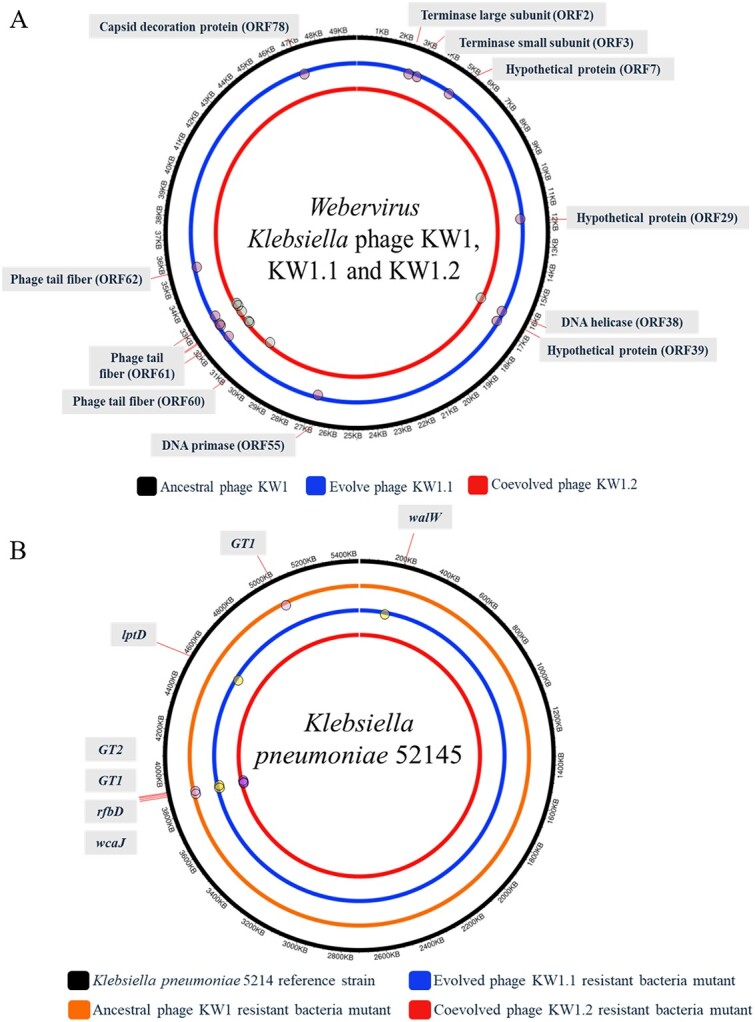
Whole genome representation and SNPs identification found in phage and phage-resistant bacteria mutants. (A) Whole genome representations of *Webervirus Klebsiella* phage. The outermost to innermost circles are ancestral phage KW1 evolved phage KW1.1 and co-evolved phage KW1.2 respectively. The SNPs location found in evolved and co-evolved phages are indicated by the circle-shaped icon. (B) Whole genome representations of Klebsiella pneumonia strain 52 145 bacteria. The outermost to innermost circles are *K. pneumoniae* strain 52 145, ancestral phage KW1 resistant bacteria mutants, evolved phage KW1.1 resistant bacteria mutants and co-evolved phage KW1.2 resistant bacteria mutants. The SNPs location found in all phage-resistant bacteria mutants are indicated by the circle-shaped icon.

Despite sharing similar genome characteristics, both the evolved phage KW1.1 and the co-evolved phage KW1.2 were found to be carrying mutations in different phage genome coding regions compared to the ancestral phage (Fig. 4A). The full details of SNP mutational changes at the nucleotide and amino acid levels can be found in Supplementar Table S3. From the results, all of the mutations identified in both the evolved phage KW1.1 and the co-evolved phage KW1.2 were those of SNP mutations.

For the evolved phage KW1.1, a total of 11 unique point mutations were observed across different putative functional phage genes. In particular, 10 out of the 11 point mutations identified were missense point mutations resulting in different amino acids being encoded, and 1 out of the 11 point mutations identified was a silent point mutation, where there was no change in the encoded amino acids. Of the 10 identified missense point mutations, 7 of the missense point mutations were found in putative functional phage genes, such as terminase large subunit (ORF2), terminase small subunit (ORF3), DNA helicase (ORF38), DNA primase (ORF55), phage tail fibers (ORF60 and 61) as well as capsid decoration protein (ORF78). The other 3 missense point mutations were identified in putative functional phage genes that encode hypothetical proteins (ORF7, ORF29, and ORF39). On the other hand, the one silent point mutation identified was found in the putative functional phage gene that encoded phage tail fibres (ORF62).

For the co-evolved phage KW1.2, a total of 5 unique missense point mutations were observed in 3 different putative functional phage genes. These missense point mutations were found in DNA helicase (ORF38) and phage tail fibers (ORF60 and ORF61), respectively. Meanwhile, both the evolved phage KW1.1 and the co-evolved phage KW1.2 were also found to share two common point missense mutations, which were found in the putative phage gene encoding phage tail fibre (ORF61).

### Identification of phage receptors through phage-resistant bacteria mutant whole-genome sequencing

The genomes of phage-resistant bacteria mutants were also analysed to screen for mutational changes that confer phage resistance. The results shown in Fig. 4B highlighted the mutational changes identified in phage-resistant bacteria mutants after referencing to the *K. pneumoniae* 52 145 strain. From the results, all the mutations identified were those of SNPs or indel mutations. All phage-resistant bacteria mutants were found sharing a common SNP leading to a nonsense mutation in the *wcaJ* gene. For the phage KW1–resistant bacteria mutants, SNPs leading to missense mutations were found in genes encoding for glycosyltransferase group 1 (herein referred to as the *GT1* gene) and group 2 family protein (herein referred to as the *GT2* gene). For phage KW1.1–resistant bacteria mutants, SNPs leading to missense and nonsense mutations were identified. Two missense mutations were found in *lptD* and *walW* genes, while 1 nonsense mutation was found in the *GT1* gene. On the other hand, the mutations found in the phage KW1.2–resistant bacteria mutants were those of indel frameshift mutations. These mutations were found in the *rfbD* gene as well as the *GT2* gene. The details of mutational changes at the nucleotide and amino acid levels can be found in Supplementary Table S2.

## Discussion

### Phage physiology and infectivity differ by phage training models

The constant adaptation and re-adaptation evolutionary relationship between phage and bacteria is one of the main factors that drives the diverse microbial communities in the natural environments [2]. Within the context of phage therapy, the dynamic antagonistic relationship between phage and bacteria presents an opportunity to harness phage evolutionary potential that can further benefit the therapeutic use of phages. Phage training is a unique tool that has been shown to leverage the suitability of phage candidates to better adapt and infect target bacteria hosts [4, 5]. However, currently reported phage training models, namely evolutionary and co-evolutionary phage training, presented conflicting phenomena. In particular, both phage training models have been claimed able to effectively suppress the emergence of phage-resistant bacteria, which raises the question of which phage training model should be used. Therefore, this study was set out to systematically compare and determine the direct influences of both evolutionary and co-evolutionary phage training models towards phage physiology and infectivity that would improve phage therapeutic efficacy.

From the results, both evolved and co-evolved phage populations were found to exert time-dependent improved suppression of bacterial growth compared to the ancestral phage KW1. The improved suppression activity exerted by evolved and co-evolved phages contradict the observed phage population dynamics. Despite the overall improved suppression of bacteria growth, the evolved phage KW1.1 and the co-evolved phage KW1.2 were found to have a reduced phage adsorption rate and varying burst size. Phage features (e.g. phage adsorption and burst size) are correlated with the virulence of phages [13]. Phage candidates with high phage adsorption and burst size are often selected as preferred therapeutic phage candidates, as the elimination of the target bacteria host in a short time period is warranted [13, 14]. The contradicting phage population dynamics and phage virulence observed in this study highlighted the complex phage–host interactions that extend beyond phage population dynamic parameters. Although limited studies were found assessing the correlational relationship between phage population dynamics and virulence, factors such as bacteria physiology, spatial heterogeneity, and configuration have been reported as possible factors that indirectly impact phage population dynamics as well as virulence [15–17]. In this study, it is postulated that the physiology of bacteria have responded differently under the exposure of evolved or co-evolved phages, subsequently affecting phage population dynamics and virulence. Further studies are still needed to assess bacterial physiology and its correlational relationship towards phage population dynamics and phage virulence.

### Phage functionally associated genes readily evolve during evolutionary and co-evolutionary phage training

To investigate the molecular mechanisms that contribute toward improved phage phenotypic features, the genomes of ancestral phage KW1, evolved phage KW1.1, and co-evolved phage KW1.2 were sequenced and analysed. All phages in the study share similar genome features such as genome length, guanine-cytosine content, and number of ORFs. However, main differences between these phages can be observed in the unique and common point missense mutations found in different phage genome regions. Particularly, the evolved phage KW1.1 and the co-evolved phage KW1.2 were found to carry mutations in putative phage genes that are functionally associated with phage tail structures or DNA replication and packaging.

The mutations identified in phage tail fibers came as expected. Phage tail fibers constitute a part of the phage tail structure that is responsible for the recognition and attachment of bacterial cell surface receptors, which is crucial for initiation of phage infection [18, 19]. Specifically, the display of phage resistance by bacteria through molecular changes in bacterial cell surface receptors impeded the functional recognition and attachment of phage tail fibers, a phenomenon commonly reported in phage resistance studies [1, 9, 20]. Therefore, to overcome bacterial phage resistance, phages may readily evolve their tail fibers to regain attachment to the previously phage-resistant bacteria.

Apart from phage tail fibers, unique mutations were also found in phage genes functionally associated with DNA replication, DNA packaging, and phage head stabilization. Phage DNA replication and packaging remain an important step during the phage replication process to ensure successful generation of phage progeny [21, 22]. Our findings are consistent with previous studies which also investigated the role of phage functional genes such as terminase, capsid and scaffold protein affecting phage adsorption, and burst sizes as well as infectivity [23–25]. In this study, we postulated that the mutations identified in the evolved phage KW1.1 and the co-evolved phage KW1.2 may have contributed to the improvement in phage infectivity compared to the ancestral phage KW1. It should be noted that the mutational changes identified in putative functionally associated phage genes contributing to altered phage phenotype and virulence are yet to be validated. Further studies about phage genome editing and complementation assays are still needed.

### Phage-resistant bacteria mutants using lipopolysaccharide membrane protein to evade phage irreversible attachment

In this study, the phage-resistant bacteria mutants were observed to have SNP and indel mutations in genes involved in biosynthesis of two major bacteria cell surface proteins, namely colanic acid capsule and lipopolysaccharide (LPS). Colanic acid is an exopolysaccharide that is commonly produced by *Enterobacteriacea* and forms a capsule surrounding the bacteria [26]. Meanwhile, LPS is a polysaccharide that forms an integral component of the gram-negative bacteria outer membrane structure [27]. Both the colanic acid capsule and LPS have been previously reported as common phage receptors utilized by many phages [28–30]. In this study, the mutational changes observed in both the colanic acid capsule and LPS proteins revealed the possible reversible attachment of the phage to the colanic acid capsule first, followed by irreversible attachment to LPS to initiate infection (Fig. 3C-E).

Interestingly, the majority of unique mutations identified in the ancestral, evolved, or co-evolved phage-resistant bacteria mutants were found in genes involved in biosynthesis of LPS proteins. Dependent on the phage encountered, these mutations would be present in different LPS biosynthesis related genes. This observed phenomenon highlights the finding that the alteration of LPS protein by bacteria is crucial to resist phage infection. Additionally, the mutations found in different genes involved in LPS biosynthesis suggest that both the evolved and co-evolved phages adhere differently to the LPS membrane protein for its irreversible attachment. This is also evident from the mutations identified in the phage tail fiber gene of evolved and co-evolved phages (ORF60 and ORF61), in which unique SNP mutations are located at different nucleotide positions even within the same phage tail fiber gene. Future studies involving complementation assays are needed to validate the putative functional role of the mutated phage tail fiber genes and its correlation in recognizing the colanic acid capsule and LPS proteins.

### The fluctuated phage sensitivity and resistance dynamics of phage-resistant bacteria mutants

Although improved suppressions of bacteria growth were observed in both evolved and co-evolved phages, the emergence of phage-resistant bacteria mutants could still be observed during phage training. These phage-resistant bacteria mutants were found to have varying levels of resistance against both evolved and co-evolved phage populations. Two main evolutionary concept models, namely arms race dynamics (ARD) and fluctuating-selection dynamics (FSD) have been used thus far to describe the antagonistic adaptations and counter-adaptations between bacteria and phage [31]. For ARD, the accumulative selection pressure favours the evolution of a particular bacterial or phage genotype that confers monotonous increased resistance or infectivity to multiple phage or bacteria genotypes over a long period of time [7, 31]. In contrast, for FSD, the evolution of the bacterial or phage genotype is time independent (also known as negative-frequency dependent) and relies on the presence of a phage or bacteria genotype occurring at the given time point, thereby resulting in temporal fluctuating phage infectivity or phage resistance dynamic [4, 7, 32]. In this study, the observed varying resistance dynamics of phage-resistant bacteria mutants against the ancestral phage, evolved and co-evolved phage populations highlighted the FSD-like dynamics pattern. Interestingly, the FSD-like dynamics of both evolved and co-evolved phage-resistant bacteria presented an interesting phenomenon. Particularly, the evolved phage-resistant bacteria mutants were found to have monotonous increased phage resistance against evolved phage populations whereas the majority of co-evolved phage-resistant bacteria mutants exhibited a low level of phage resistance. It is postulated that the evolved phage-resistant bacteria mutants may have accumulated more mutational changes compared to the co-evolved phage-resistant mutants. Indeed, the evolved phage and the evolved phage-resistant bacteria mutants were found to harbour more genome mutations compared to its co-evolved counterpart. It is also worthwhile to note that the current mutations identified in both phage and phage-resistant bacteria mutants were from single-colony isolates, which may underestimate the actual genomic representations at population level. Therefore, population sequencing of evolved and co-evolved phage and its phage-resistant bacteria mutant counterparts are needed to assess the mutational change at population level.

### The phage resistance barrier dictates the evolution of evolved and co-evolved phages

Both phenotypic and genomic data illustrated in our study highlighted that evolutionary and co-evolutionary phage training can improve phage infectivity. Although it is unclear how these different phage training approaches resulted in different phage traits observed in our study, it is postulated that the type of phage resistance encountered during phage training may play an important role [33–35].

**Figure 5:**
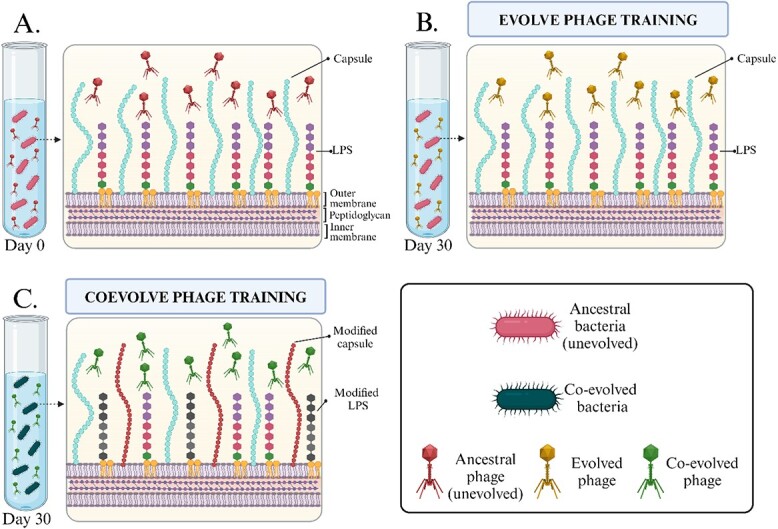
Proposed molecular changes in bacteria membrane protein during evolutionary and co-evolutionary phage training. (A) Proposed molecular responses of bacterial membrane proteins encountered by ancestral phage before phage training. (B) Proposed molecular responses of bacterial membrane proteins within evolutionary phage training model, where changes in bacterial membrane remain constant from day 0 to day 30. (C) Proposed molecular responses of bacterial membrane proteins where dynamic changes had occurred during co-evolutionary phage training, resulting in modified membrane protein (depicted with color change). Created with BioRender.com.

In the evolutionary phage training, phage is directed to continuously evolve to infect the same bacteria. Although the phage will encounter resistance by bacteria, the phage resistance mechanisms are postulated to be the same throughout the course of phage evolution (Fig. 5A and B). As such, phages could readily evolve relevant phage traits to overcome this resistance barrier. This process is reflective through the mutational changes identified in the evolved phage KW1 putative tail fibre genes and also other putative function–related genes involved in DNA replication and packaging, as well as phage head stability.

Differently, in the co-evolutionary phage training, both the phage and bacteria are subjected to continuous co-evolution. In this approach, the evolution of the co-evolved phage is directly influenced by the presence of co-evolved bacteria. Here, both the co-evolved phage and bacteria are constantly adapting to counteract each other. It is hypothesized that the co-evolved bacteria during the co-evolutionary training display fluctuating dynamic of phage resistance mechanisms to escape the predation of co-evolved phage (Fig. 5C). This is evident through the observed FSD-like resistance dynamics of co-evolved phage-resistant bacteria mutants but also the mutations identified in co-evolved phage KW1.2, where the majority of the mutations were limited to putative phage tail fibre genes.

Collectively, our results favour the use of co-evolved phage training model for two reasons. First, the co-evolved phage training model can train phages that display improved bacteria growth suppression and delay the emergence of phage resistant bacteria. Second, phage-resistant bacterial mutants that are likely to arise after infections by co-evolved phages are more susceptible to the trained co-evolved phages from the past, present, and future, thus reduce the risk of phage resistance in bacteria. It is also worthwhile to note that whilst contrasting effects between evolutionary and co-evolutionary phage training models were observed in our study, our current observations are limited to one phage–bacteria model (i.e. *Klebsiella* phage and *K. pneumoniae* bacteria). Therefore, it is recommended that different phage–bacteria models encompassing different bacteria models (i.e. gram positive and gram negative) should be evaluated to truly validate the repeatability and contrasting effects of different phage training models.

## Conclusions

Phage training is a powerful evolutionary approach that can improve the therapeutic efficacy of phages. In this study, both evolutionary and co-evolutionary phage training models were applied towards phages infecting *K. pneumoniae*. After training, both evolved and co-evolved phages were found to have improved phage infectivity. The improved phage infectivity displayed by both evolved and co-evolved phages correlated well with the mutations identified in different putative functionally associated phage genes, but also the mutations identified in phage-resistant bacteria mutants. It is hypothesized that the differences observed in both evolved and co-evolved phage traits are due to the encountered phage resistance barrier imposed by bacteria during phage training. Additionally, phage resistance dynamics of the evolved and co-evolved phage-resistant bacteria mutants highlighted the advantage of using co-evolutionary phage training model for phage training. Future studies involving complementation assays, population sequencing as well as assessment of bacteria physiology are still needed to validate the correlations of different phage training models with phenotypic and genomic characteristics of both phage and bacteria.

## Supplementary Material

Supporting_information_ycae082
