# Supplementary material for: Evolutionary and co-evolutionary phage training approaches enhance bacterial suppression and delay the emergence of phage resistance
Source: ISME Commun. 2024 Jun 12;4(1):ycae082. doi: 10.1093/ismeco/ycae082 (PMC11234896; doi:10.1093/ismeco/ycae082)
Supplement: Supporting_information_ycae082 [file supporting_information_ycae082.docx]

**Supporting Information**

**Evolutionary and coevolutionary phage training approaches enhance bacterial suppression and delay the emergence of phage resistance**

Lyman Ngiam ^a^, Karen D. Weynberg* ^a^, Jianhua Guo* ^a^

^a^ Australian Centre for Water and Environmental Biotechnology, University of Queensland, Brisbane, Australia

*These authors have contributed equally to this work

Corresponding author: [jianhua.guo@uq.edu.au](mailto:jianhua.guo@uq.edu.au), Gehrmann Laboratories Building, The University of Queensland, Research Rd, St Lucia QLD 4067, +61 7 3346 3222.

**This file includes:**

Supplementary Text 1

Supplementary Tables 1 to 3

Supplementary Figures 1 to 24

**Text S1. Processing raw Illumina and Nanopore data for hybrid assembly using Unicycler**

Raw reads received were trimmed and filtered by using Trimmomatic (Bolger et al., 2014) to remove adaptor sequences and reads less than 36 bp when a sliding window of 4 bp and minimum Phred score of 30 was applied. Meanwhile, raw data generated from Nanopore sequencing in Fast5 format were basecalled and converted in Fastq format using Guppy neural network basecalling software (Wick et al., 2019).

**Table S1.** List of genome features of ancestral, evolved and coevolved phage

| Name | Type | Start | Stop | Length |
| --- | --- | --- | --- | --- |
| Putative portal protein | CDS | 1 | 1191 | 1191 |
| Putative terminase large subunit | CDS | 1237 | 2838 | 1602 |
| Putative terminase small subunit | CDS | 2848 | 3372 | 525 |
| Hypothetical protein | CDS | 3451 | 3639 | 189 |
| Hypothetical protein | CDS | 3717 | 3980 | 264 |
| Hypothetical protein | CDS | 3974 | 4168 | 195 |
| Hypothetical protein | CDS | 4296 | 4529 | 234 |
| Hypothetical protein | CDS | 4517 | 4753 | 237 |
| Hypothetical protein | CDS | 4823 | 5398 | 576 |
| Hypothetical protein | CDS | 5395 | 5724 | 330 |
| Hypothetical protein | CDS | 5718 | 5942 | 225 |
| Hypothetical protein | CDS | 5975 | 6604 | 630 |
| Hypothetical protein | CDS | 6680 | 7168 | 489 |
| Hypothetical protein | CDS | 7168 | 7386 | 219 |
| Hypothetical protein | CDS | 7466 | 7651 | 186 |
| Hypothetical protein | CDS | 7648 | 7893 | 246 |
| Hypothetical protein | CDS | 7893 | 8195 | 303 |
| Hypothetical protein | CDS | 8192 | 8518 | 327 |
| Hypothetical protein | CDS | 8588 | 8908 | 321 |
| Hypothetical protein | CDS | 8919 | 9131 | 213 |
| Hypothetical protein | CDS | 9200 | 9424 | 225 |
| Putative anticodon nuclease activator family protein | CDS | 9494 | 9652 | 159 |
| Hypothetical protein | CDS | 9796 | 9921 | 126 |
| Hypothetical protein | CDS | 9928 | 10512 | 585 |
| Hypothetical protein | CDS | 10579 | 10824 | 246 |
| Hypothetical protein | CDS | 10918 | 11019 | 102 |
| Hypothetical protein | CDS | 11167 | 11349 | 183 |
| Hypothetical protein | CDS | 11539 | 11733 | 195 |
| Hypothetical protein | CDS | 11730 | 11969 | 240 |
| Hypothetical protein | CDS | 11969 | 12352 | 384 |
| Putative methyl-directed repair DNA adenine methylase | CDS | 12352 | 13044 | 693 |
| Hypothetical protein | CDS | 13117 | 13344 | 228 |
| DNA cytosine methyltransferase | CDS | 13341 | 14033 | 693 |
| Hypothetical protein | CDS | 14106 | 14309 | 204 |
| Hypothetical protein | CDS | 14306 | 14488 | 183 |
| Hypothetical protein | CDS | 14489 | 14926 | 438 |
| Hypothetical protein | CDS | 14923 | 15021 | 99 |
| Putative DNA helicase | CDS | 15052 | 16620 | 1569 |
| Hypothetical protein | CDS | 16624 | 17085 | 462 |
| Putative u-spanin | CDS | 17155 | 17586 | 432 |
| Putative lysin | CDS | 17583 | 18065 | 483 |
| Putative holin | CDS | 18067 | 18282 | 216 |
| Putative nucleoside triphosphate hydrolase | CDS | 18408 | 18989 | 582 |
| Putative polynucleotide kinase | CDS | 18986 | 19474 | 489 |
| Putative sigma-70 region 4 domain containing protein | CDS | 19512 | 20642 | 1131 |
| Hypothetical protein | CDS | 20738 | 20989 | 252 |
| Hypothetical protein | CDS | 20986 | 21222 | 237 |
| Hypothetical protein | CDS | 21287 | 21523 | 237 |
| Putative DNA adenine methyltransferase | CDS | 21527 | 22258 | 732 |
| Hypothetical protein | CDS | 22261 | 22539 | 279 |
| VRR-NUC domain-containing protein | CDS | 22613 | 22942 | 330 |
| Putative ATP dependent helicase | CDS | 23020 | 25056 | 2037 |
| Putative HNH endonuclease | CDS | 25053 | 25565 | 513 |
| Transcriptional regulator | CDS | 25657 | 26058 | 402 |
| DNA primase | CDS | 26170 | 27093 | 924 |
| Hypothetical protein | CDS | 27297 | 27458 | 162 |
| PD-(D/E)XK nuclease-like domain-containing protein | CDS | 27589 | 28635 | 1047 |
| ERF family protein | CDS | 28695 | 29351 | 657 |
| Putative single-stranded DNA binding protein | CDS | 29388 | 29849 | 462 |
| Putative tail fiber protein | CDS | 29972 | 32488 | 2517 |
| Hypothetical protein | CDS | 32516 | 33472 | 957 |
| Putative tail fiber protein | CDS | 33474 | 36947 | 3474 |
| Putative tail assembly protein | CDS | 37035 | 37637 | 603 |
| Putative tail assembly protein | CDS | 37612 | 38349 | 738 |
| Minor tail protein | CDS | 38351 | 39103 | 753 |
| Putative tail protein | CDS | 39173 | 39517 | 345 |
| Putative tail tape measure protein | CDS | 39520 | 42486 | 2967 |
| Tape measure chaperone | CDS | 42523 | 42690 | 168 |
| Tail assembly chaperone | CDS | 42663 | 42758 | 96 |
| Putative tail assembly chaperone protein | CDS | 42837 | 43235 | 399 |
| Putative major tail protein | CDS | 43240 | 43896 | 657 |
| Putative tail-to-head joining protein | CDS | 43990 | 44496 | 507 |
| Putative neck protein | CDS | 44411 | 44848 | 438 |
| Putative head-to-tail connector complex protein | CDS | 44841 | 45218 | 378 |
| Putative head-to-tail connector complex protein | CDS | 45224 | 45640 | 417 |
| Hypothetical protein | CDS | 45694 | 45981 | 288 |
| DUF2184 domain-containing protein | CDS | 46073 | 47032 | 960 |
| Putative capsid decoration protein | CDS | 47147 | 47656 | 510 |
| Putative major capsid protein | CDS | 47708 | 48838 | 1131 |
| Putative minor capsid protein | CDS | 48838 | 49608 | 771 |
| Putative portal protein | CDS | 49595 | 49714 | 120 |

**Table S2.** Summary of genomic mutational changes observed in phage-resistant bacteria mutants. * The mutational change at nucleotide level is displayed in red, bold, underlined font.

| Phage | Gene / protein | Mutational change* | Functional role of gene |
| --- | --- | --- | --- |
| vB_KpS_KW1 | *wcaJ gene* | SNP; nonsense  (CAG > **T**AG ) | Glycosyltransferase, Initiating enzyme of colanic acid synthesis |
|  | Glycosyltransferase family 2 protein | SNP; missense  (ACG > A**U**G) | Glycosyltransferase, Involved in biosynthesis of LPS-O antigen |
|  | Group-1 glycosyltransferase | SNP; missense  (AAA > **C**AA) | Glycosyltransferase, Involved in biosynthesis of LPS-O antigen |
| vB_KpS_KW1.1 | *wcaJ gene* | SNP; nonsense  (CAG > **T**AG ) | Glycosyltransferase, Initiating enzyme of colanic acid synthesis |
|  | *lptD gene* | SNP; missense  (GGC > **A**GC ) | Involved with LPS assembly in outer membrane protein |
|  | *walW gene* | SNP; missense  (ACG > A**T**G) | Involved with LPS synthesis |
|  | Glycosyltransferase, group 2 family protein | SNP; nonsense  (CAA > **T**AA) | Glycosyltransferase, Involved in biosynthesis of LPS-O antigen |
| vB_KpS_KW1.2 | *wcaJ gene* | SNP; nonsense  (CAG > **T**AG ) | Lipopolysaccharide (LPS) core biosynthesis |
|  | *rfbD gene* | Indel; frameshift  (deletion; **-A**) | Synthesizes one of the precursors for the O antigen of LPS |
|  | Glycosyltransferase, group 2 family protein | Indel; frameshift  (insertion; **+A**) | Glycosyltransferase, Involved in biosynthesis of LPS-O antigen |

**Table S3.** Summary of mutations identified in phage KW1.1 and phage KW1.2 in comparison to unevolved phage KW1.

| **Unique mutations identified in EVOLVED PHAGE KW1.1** | | | | | |
| --- | --- | --- | --- | --- | --- |
| ORF | Gene | Mutation | Nucleic acid change (position) | Amino acid change | Functional role of gene |
| 2 | Terminase large subunit | Missense | GGC > AGC (2514) | Gly > Ser | Endonuclease that cuts the viral genome from the concatemer to initiate and to end the packaging reaction |
| 3 | Terminase small subunit | Missense | ATC > GTC (2955) | IIe > Val | Initiate packaging of the viral genome |
| 7 | Hypothetical protein | Missense | ACG > GCG (4705) | Thr > Ala | Unknown |
| 29 | Hypothetical protein | Missense | CCT > TCT (11784) | Pro > Ser | Unknown |
| 38 | Putative DNA helicase | Missense | GGG > TGG (16276) | Gly > Trp | Unwind DNA strands for DNA replication |
| 39 | Hypothetical protein | Missense | GAC > GGC (16814) | Asp > Gly | Unknown |
| 55 | DNA primase | Missense | GGA > AGA (26761) | Gly > Arg | Catalyze the synthesis of short RNA molecules used as primers for DNA polymerases |
| 60 | Putative tail fiber protein | Missense | GGC > GAC (32016) | Gly > Asp | Responsible for the specific, albeit reversible primary attachment to host cell |
| 61 | Putative tail fiber protein | Missense | GAG > GGG (32721) | Glu > Gly | Responsible for the specific, albeit reversible primary attachment to host cell |
| 62 | Putative tail fiber protein | Silent | CGA > CGC (35658) | NA | Responsible for the specific, albeit reversible primary attachment to host cell |
| 78 | Capsid decoration protein | Missense | GGC > GTC (47154) | Gly > Val | Stabilization of phage head structure |
| **Unique mutations identified in COEVOLVED PHAGE KW1.2** | | | | | |
| 38 | Putative DNA helicase | Missense | GGC > TGC (16168) | Gly > Cys | Unwind DNA strands for DNA replication |
| 60 | Putative tail fiber protein | Missense | TTC > TTA (30271) | Phe > Leu | Responsible for the specific, albeit reversible primary attachment to host cell |
| 60 | Putative tail fiber protein | Missense | AAC > AGC (31878) | Asn > Ser | Responsible for the specific, albeit reversible primary attachment to host cell |
| 60 | Putative tail fiber protein | Missense | AAC > AAA (31990) | Asn > Lys | Responsible for the specific, albeit reversible primary attachment to host cell |
| 61 | Putative tail fiber protein | Missense | GCT > ACT (33082) | Ala > Thr | Responsible for the specific, albeit reversible primary attachment to host cell |
| **Common mutations identified in both EVOLVED PHAGE KW1.1 and COEVOLVED PHAGE KW1.2** | | | | | |
| 61 | Putative tail fiber protein | Missense | GAT > GGT (32670) | Asp > Gly | Responsible for the specific, albeit reversible primary attachment to host cell |
| 61 | Putative tail fiber protein | Missense | GGA > AGA (33187) | Gly > Arg | Responsible for the specific, albeit reversible primary attachment to host cell |


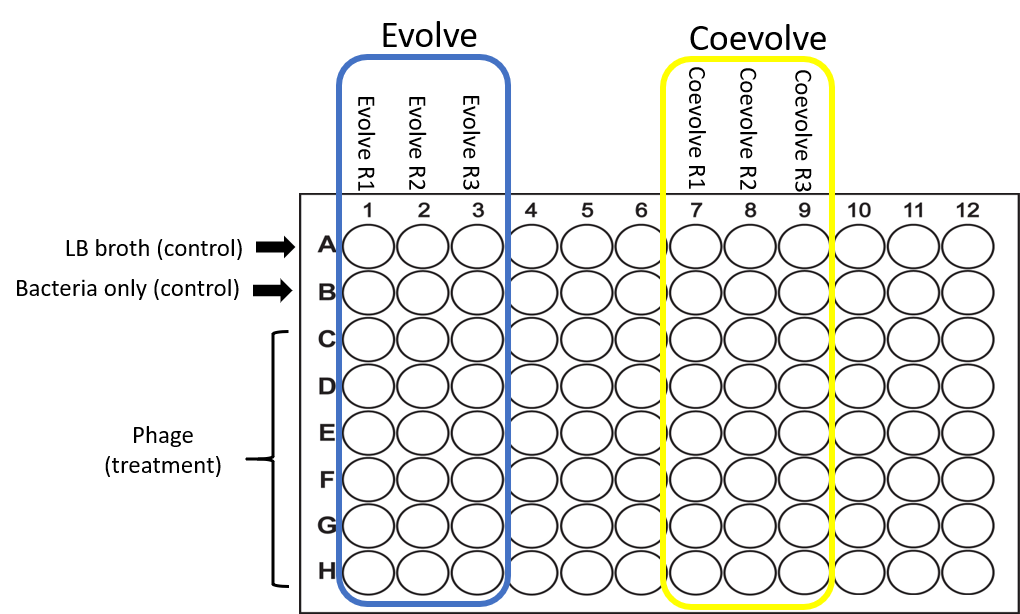


**Figure S1.** Biological triplicate design of evolutionary and coevolutionary phage training performed in 96 well plate.


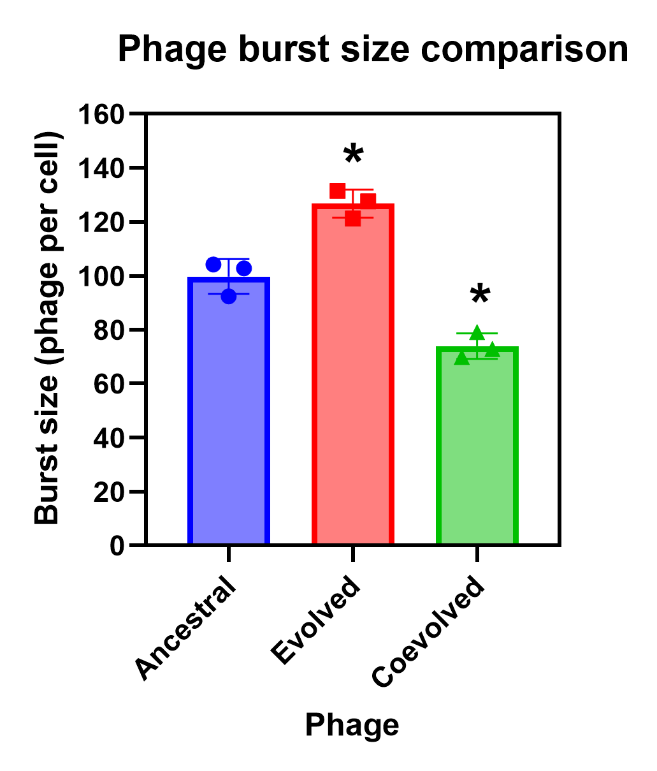


**Figure S2.** Burst size comparison of ancestral, evolved and coevolved phage. Each value is presented as mean values ± standard deviation from three independent experiments. * represents statistically significant (p <0.05).


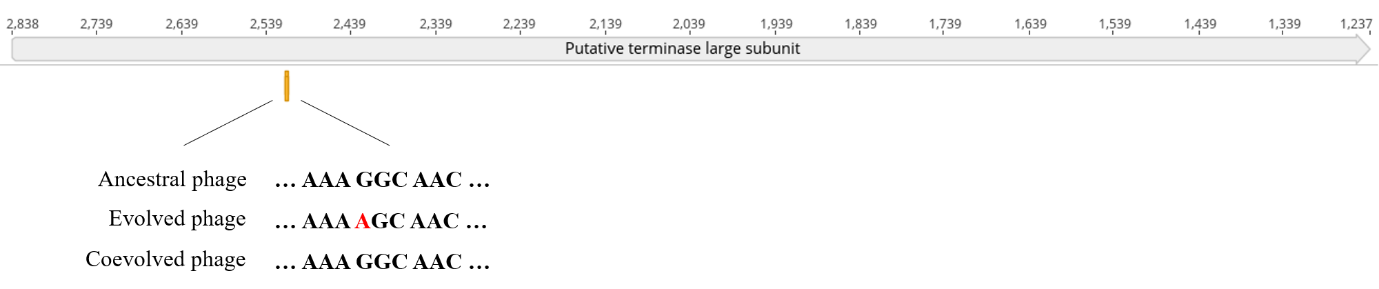


**Figure S3.** Genomic variant comparison of phage terminase large subunit (ORF2) identified in ancestral, evolved and coevolved phage. Yellow bar denotes the mutational change observed at nucleotide level. Red coloured nucleotide base denote the mutational change observed.


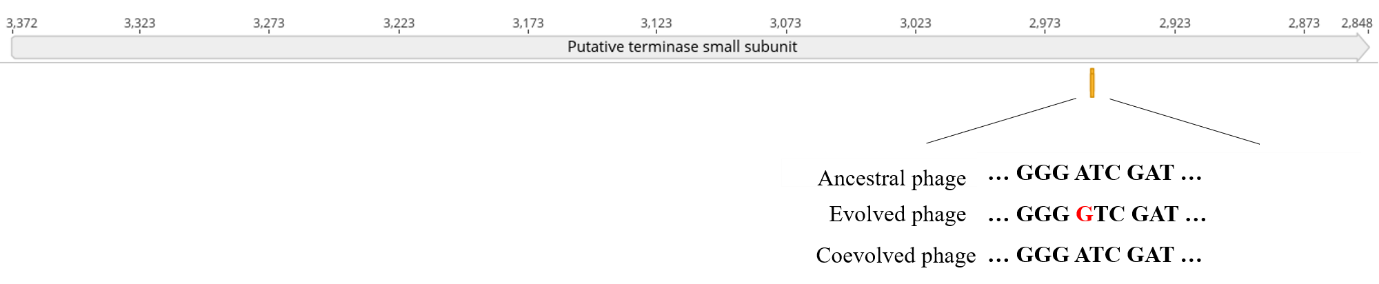


**Figure S4.** Genomic variant comparison of phage terminase small subunit (ORF3) identified in ancestral, evolved and coevolved phage. Yellow bar denotes the mutational change observed at nucleotide level. Red coloured nucleotide base denote the mutational change observed.


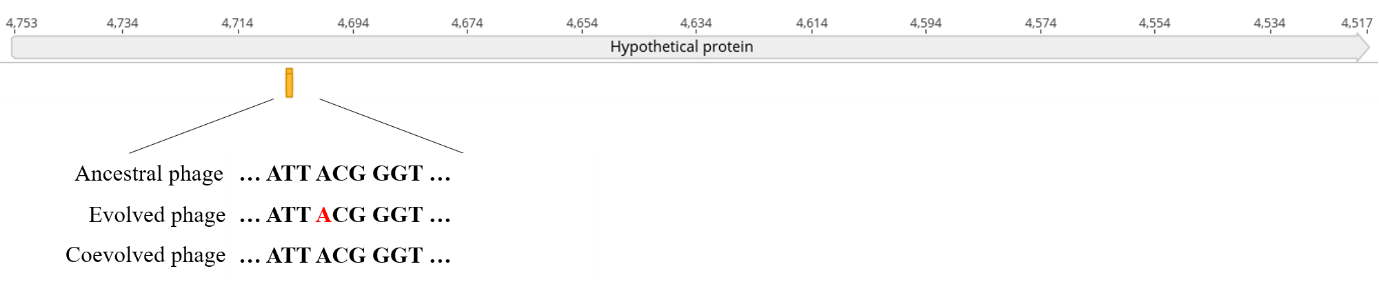


**Figure S5.** Genomic variant comparison of phage hypothetical protein (ORF7) identified in ancestral, evolved and coevolved phage. Yellow bar denotes the mutational change observed at nucleotide level. Red coloured nucleotide base denote the mutational change observed.


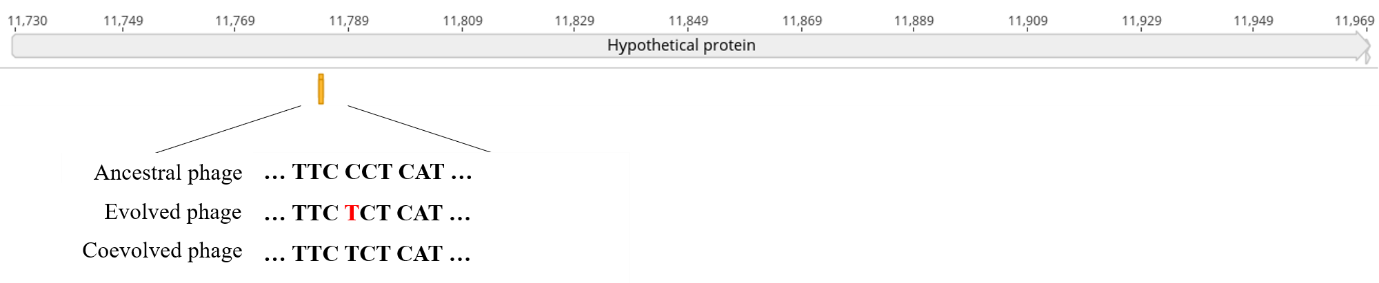


**Figure S6.** Genomic variant comparison of phage hypothetical protein (ORF29) identified in ancestral, evolved and coevolved phage. Yellow bar denotes the mutational change observed at nucleotide level. Red coloured nucleotide base denote the mutational change observed.


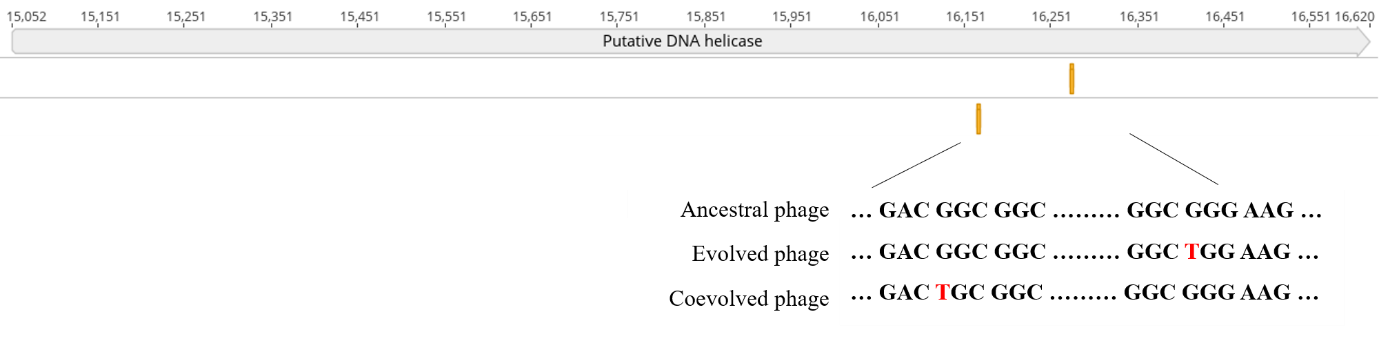


**Figure S7.** Genomic variant comparison of phage DNA helicase (ORF38) identified in ancestral, evolved and coevolved phage. Yellow bar denotes the mutational change observed at nucleotide level. Red coloured nucleotide base denote the mutational change observed.


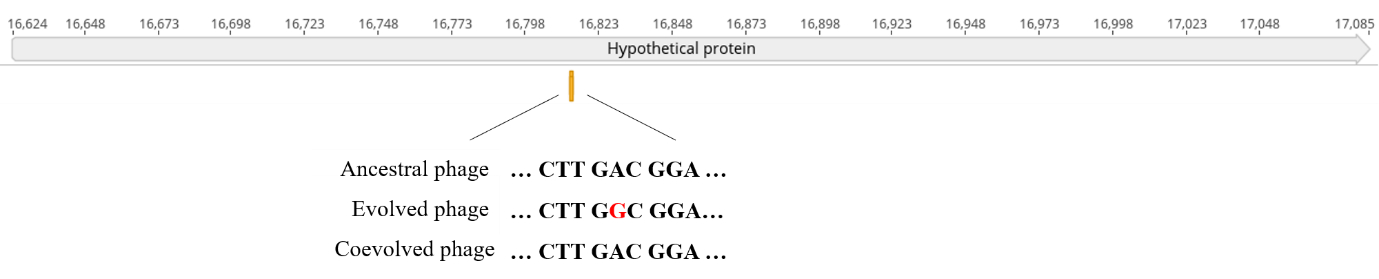


**Figure S8.** Genomic variant comparison of phage hypothetical protein (ORF39) identified in ancestral, evolved and coevolved phage. Yellow bar denotes the mutational change observed at nucleotide level. Red coloured nucleotide base denote the mutational change observed.


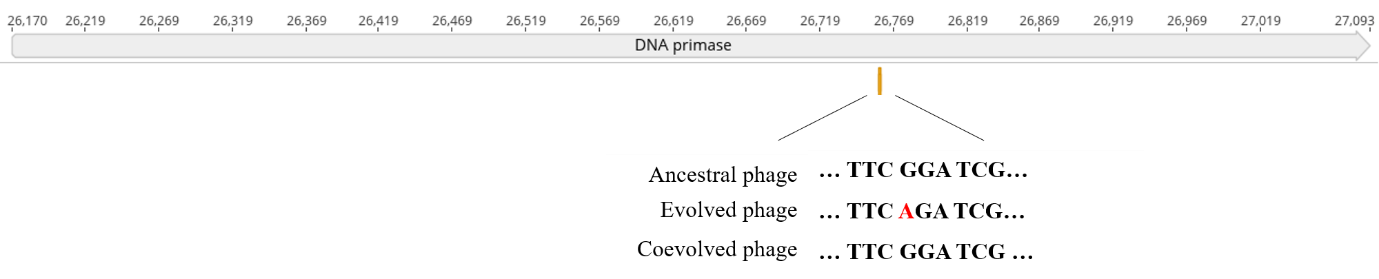


**Figure S9.** Genomic variant comparison of phage DNA primase (ORF55) identified in ancestral, evolved and coevolved phage. Yellow bar denotes the mutational change observed at nucleotide level. Red coloured nucleotide base denote the mutational change observed.


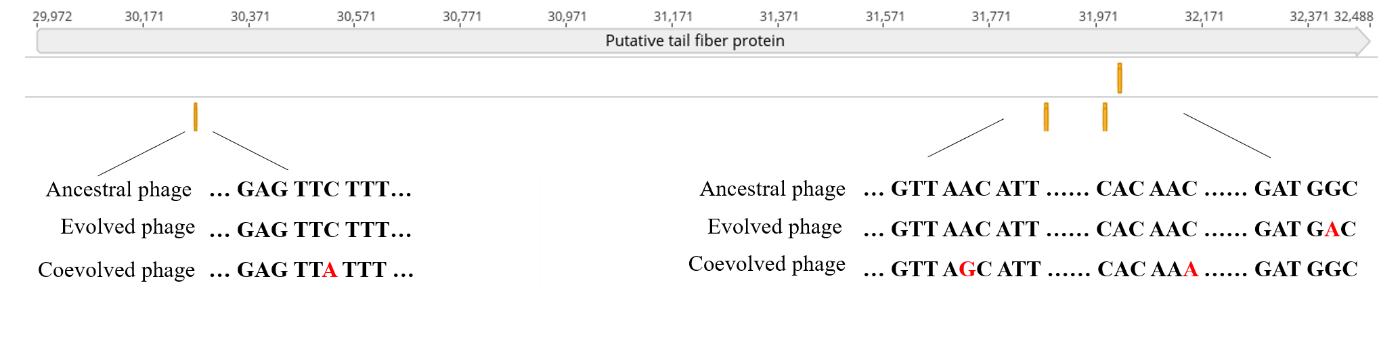


**Figure S10.** Genomic variant comparison of phage tail fiber (ORF60) identified in ancestral, evolved and coevolved phage. Yellow bar denotes the mutational change observed at nucleotide level. Red coloured nucleotide base denote the mutational change observed.


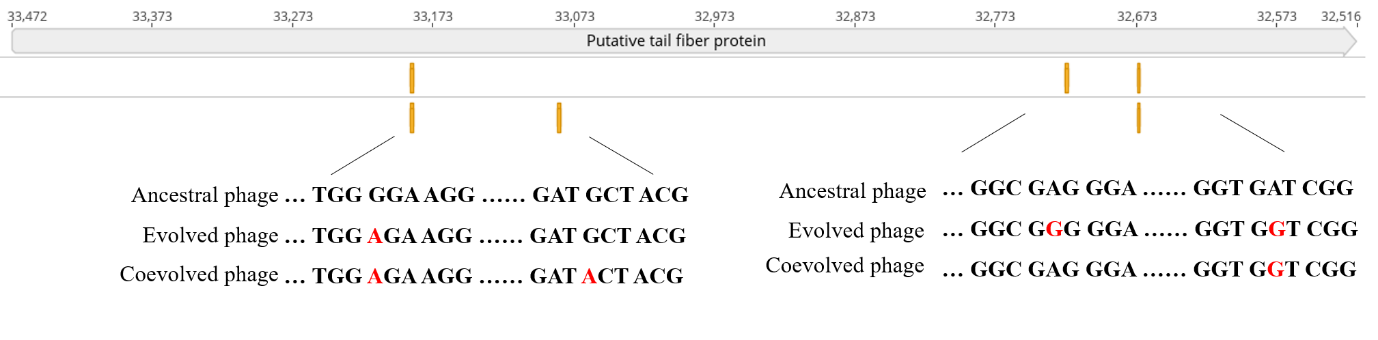


**Figure S11.** Genomic variant comparison of phage tail fiber (ORF61) identified in ancestral, evolved and coevolved phage. Yellow bar denotes the mutational change observed at nucleotide level. Red coloured nucleotide base denote the mutational change observed.


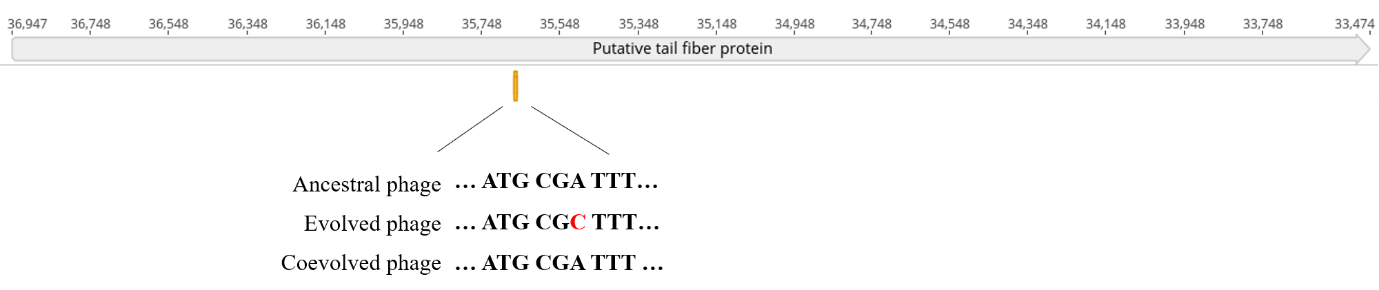


**Figure S12.** Genomic variant comparison of phage tail fiber (ORF62) identified in ancestral, evolved and coevolved phage. Yellow bar denotes the mutational change observed at nucleotide level. Red coloured nucleotide base denote the mutational change observed.


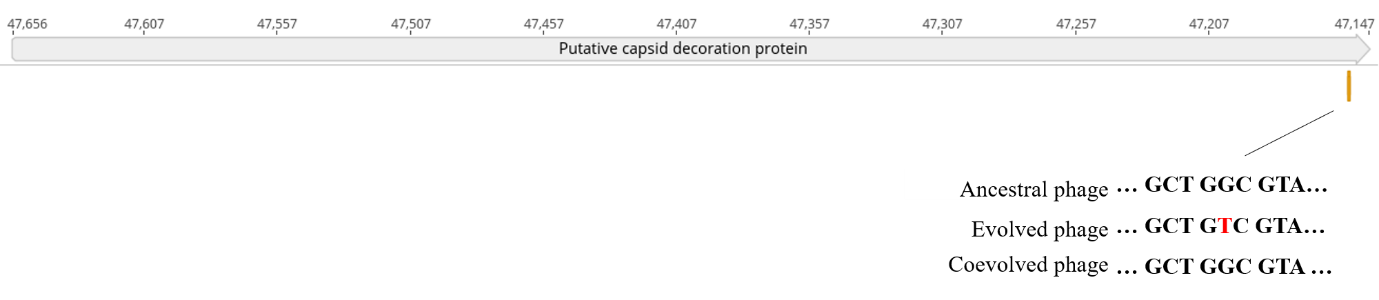


**Figure S13.** Genomic variant comparison of phage capsid decoration protein (ORF78) identified in ancestral, evolved and coevolved phage. Yellow bar denotes the mutational change observed at nucleotide level. Red coloured nucleotide base denote the mutational change observed.


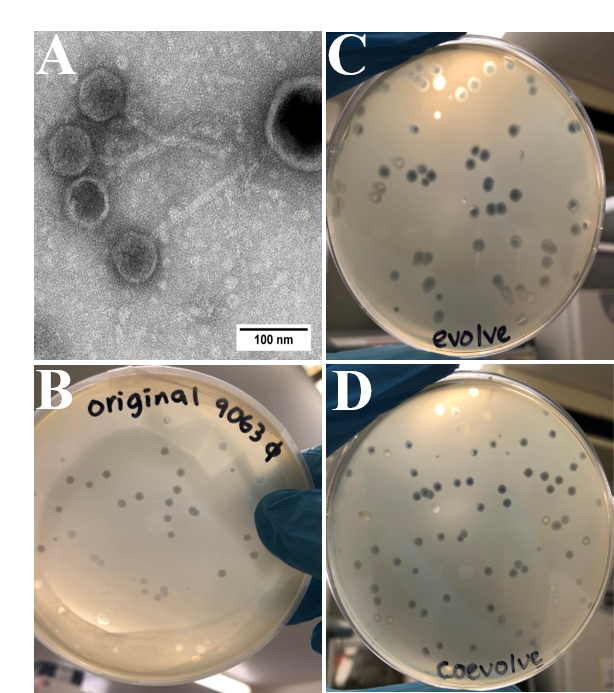


**Figure S14.** Morphology of isolated phage and the plaque morphology of unevolved, evolved and coevolved phage. (A) TEM image of isolated phage against capsule lacking *K. pneumoniae* 52145 strain; (B) Plaque morphologies of unevolved (ancestral) phage KW1; (C) Plaque morphologies of evolved phage KW1.1; and (D) Plaque morphologies of coevolved phage KW1.2.


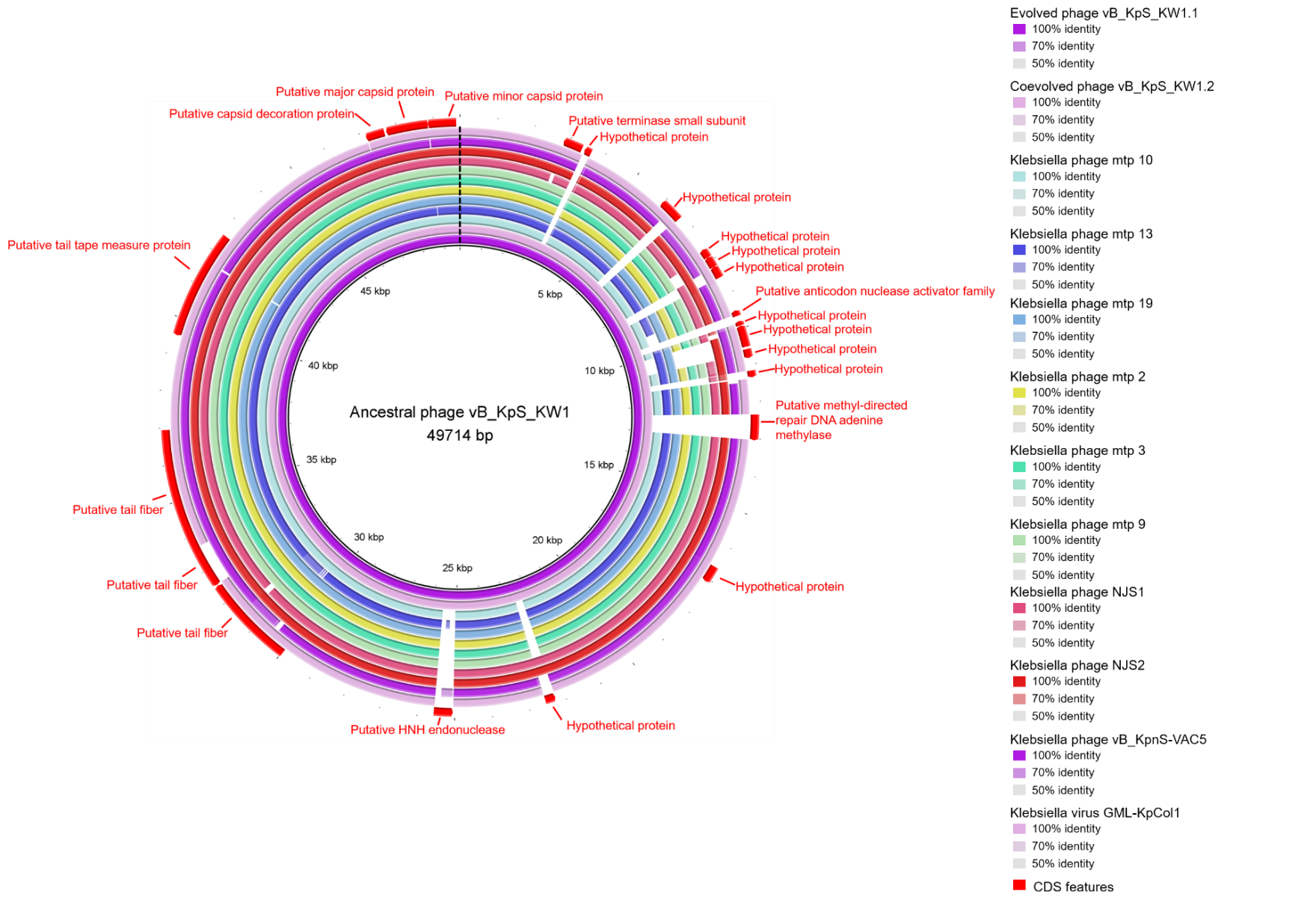


**Figure S15.** BRIG visualisation of whole genome comparison between unevolved phage KW1, evolved phage KW1.1, coevolved phage KW1.2 and top 10 closely related phage. Legend indicates which colours correspond with which phages and the shade of that colour indicates what level of similarity is observed.


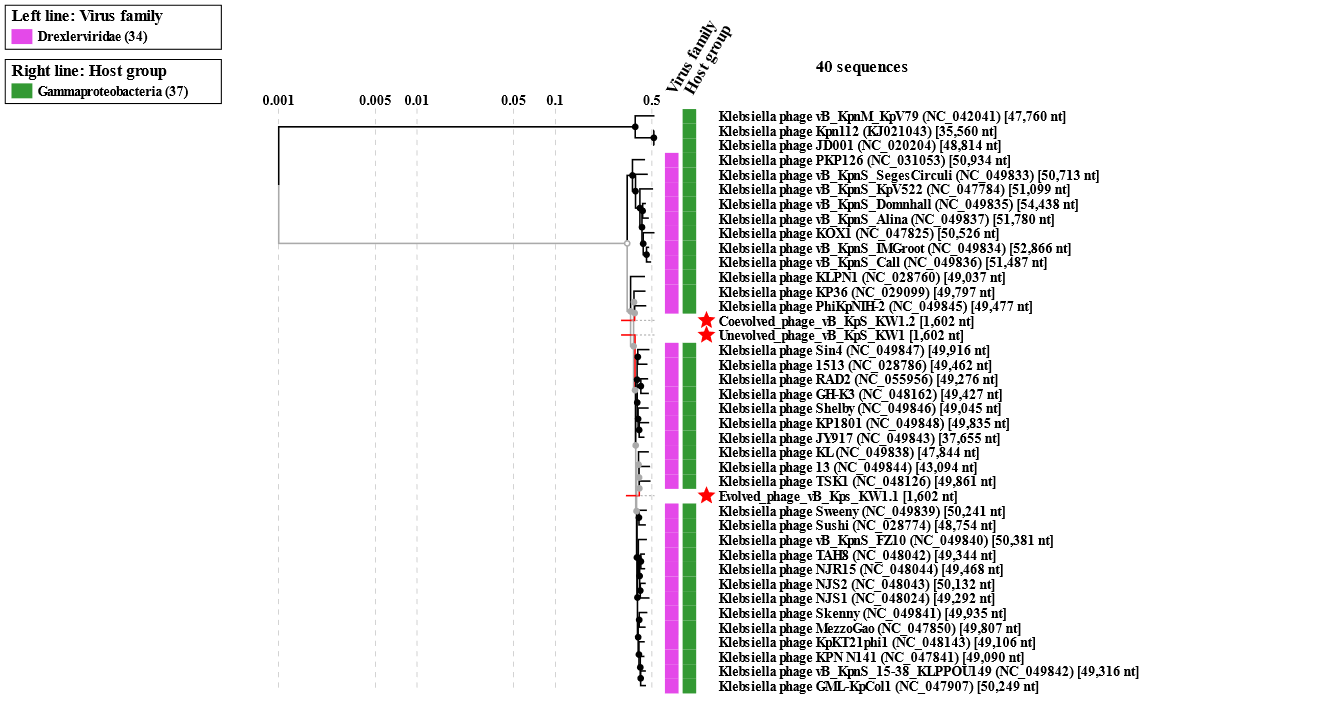


**Figure S16.** Phage phylogenetic trees constructed for protein sequences terminase large subunit using ViPTree analysis plotted on a linear scale. Phages in this study is highlighted with red star.


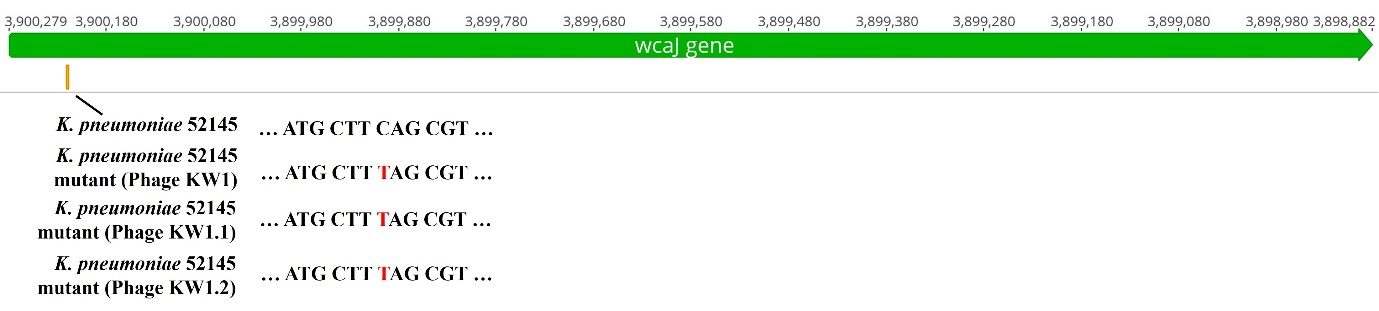


**Figure S17.** Genomic variant of *wcaJ* gene identified in ancestral, evolved and coevolved phage resistant bacteria mutants in comparison to reference strain. Yellow bar denotes the mutational change observed at nucleotide level. Red coloured nucleotide base denote the mutational change observed.


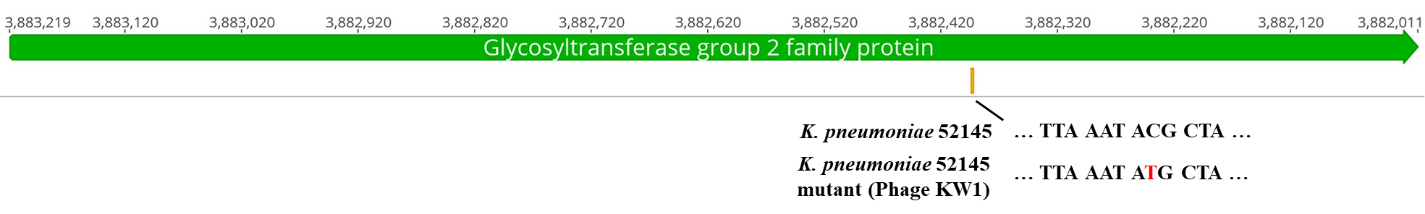


**Figure S18.** Genomic variant of glycosyltransferase group 2 family protein gene identified in ancestral phage resistant bacteria mutants in comparison to reference strain. Yellow bar denotes the mutational change observed at nucleotide level. Red coloured nucleotide base denote the mutational change observed.


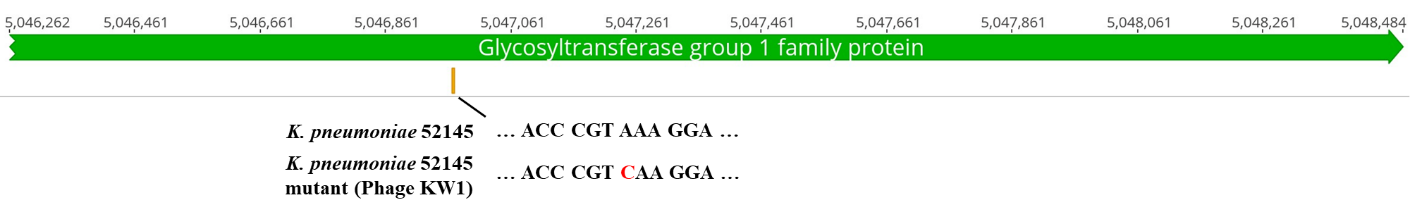


**Figure S19.** Genomic variant of glycosyltransferase group 1 family protein gene identified in ancestral phage resistant bacteria mutants in comparison to reference strain. Yellow bar denotes the mutational change observed at nucleotide level. Red coloured nucleotide base denote the mutational change observed.


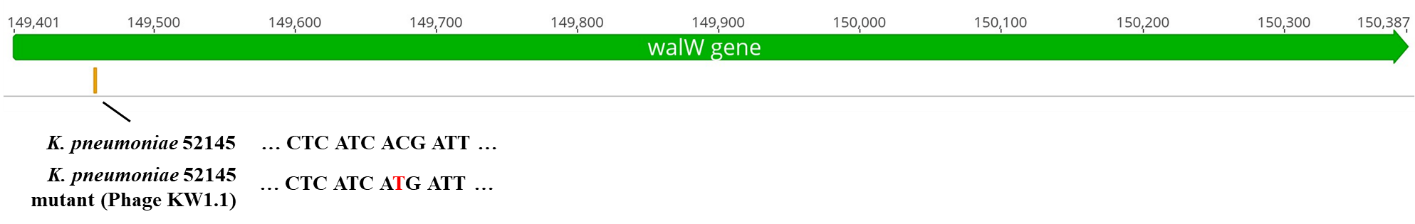


**Figure S20.** Genomic variant of *walW* gene identified in evolved phage resistant bacteria mutants in comparison to reference strain. Yellow bar denotes the mutational change observed at nucleotide level. Red coloured nucleotide base denote the mutational change observed.


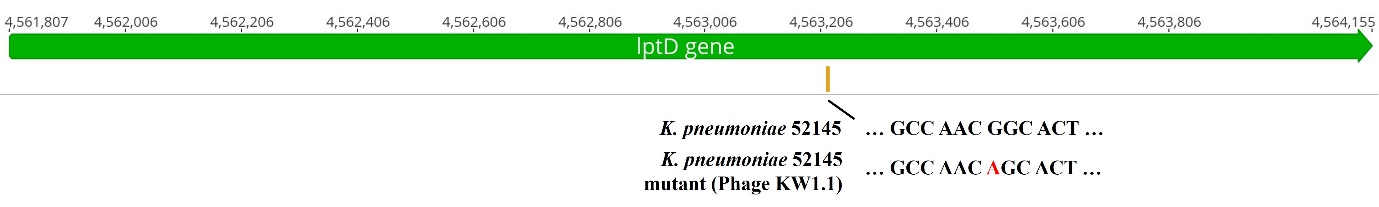


**Figure S21.** Genomic variant of *lptD* gene identified in evolved phage resistant bacteria mutants in comparison to reference strain. Yellow bar denotes the mutational change observed at nucleotide level. Red coloured nucleotide base denote the mutational change observed.


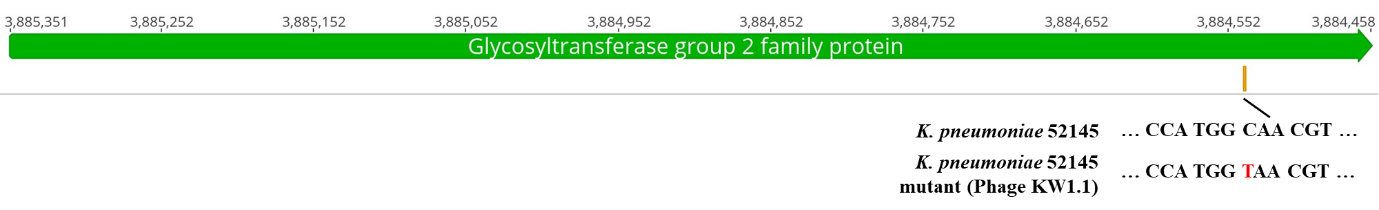
**Figure S22.** Genomic variant of glycosyltransferase group 2 family protein gene identified in evolved phage resistant bacteria mutants in comparison to reference strain. Yellow bar denotes the mutational change observed at nucleotide level. Red coloured nucleotide base denote the mutational change observed.


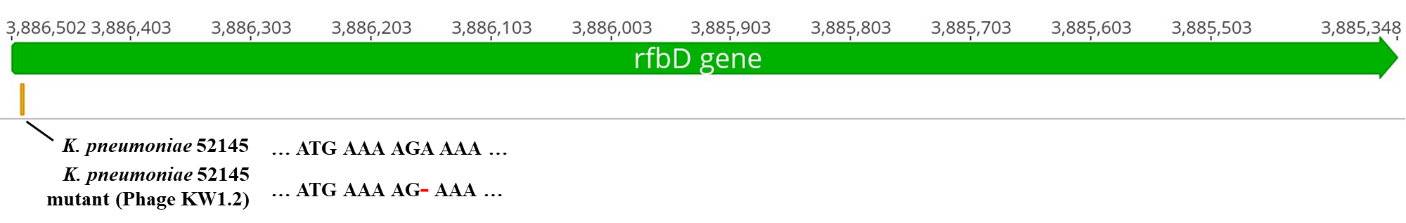


**Figure S23.** Genomic variant of *rfbD* gene identified in coevolved phage resistant bacteria mutants in comparison to reference strain. Yellow bar denotes the mutational change observed at nucleotide level. Red coloured nucleotide base denote the mutational change observed.


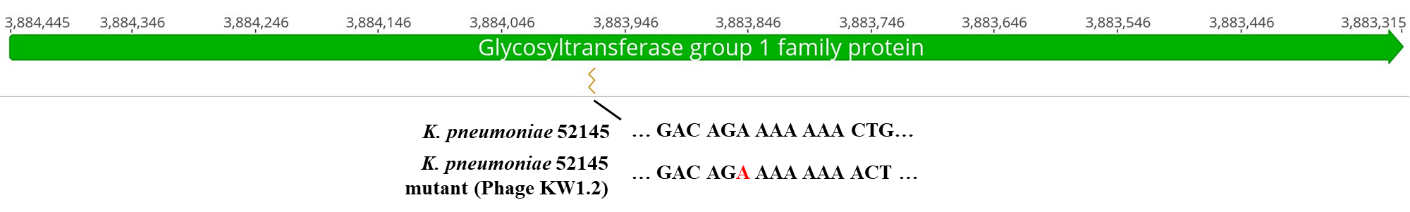


**Figure S24.** Genomic variant of glycosyltransferase group 1 family protein gene identified in coevolved phage resistant bacteria mutants in comparison to reference strain. Yellow bar denotes the mutational change observed at nucleotide level. Red coloured nucleotide base denote the mutational change observed.

REFERENCES

Bolger, A. M., Lohse, M., & Usadel, B. (2014). Trimmomatic: a flexible trimmer for Illumina sequence data. *Bioinformatics, 30*(15), 2114-2120. doi:10.1093/bioinformatics/btu170

Wick, R. R., Judd, L. M., & Holt, K. E. (2019). Performance of neural network basecalling tools for Oxford Nanopore sequencing. *Genome Biology, 20*(1). doi:10.1186/s13059-019-1727-y
